# Developmental Stage and Shape of Embryo Determine the Efficacy of Embryo Rescue in Introgressing Orange/Yellow Color and Anthocyanin Genes of *Brassica* Species

**DOI:** 10.3390/plants7040099

**Published:** 2018-11-13

**Authors:** Sreyvatey Pen, Ujjal Kumar Nath, Samnang Song, Gayatri Goswami, Ji-Hee Lee, Hee-Jeong Jung, Hoy-Taek Kim, Jong-In Park, Ill-Sup Nou

**Affiliations:** 1 Department of Horticulture, Suncheon National University, 255 Jungang-ro, Suncheon, Jeonnam 57922, Korea; pensreyvatey@gmail.com (S.P.); ujjalnath@gmail.com (U.K.N.); songsamnang@gmail.com (S.S.); gayatri_bau@yahoo.com (G.G.); gml79wjd@sunchon.ac.kr (H.-J.J.); jipark@sunchon.ac.kr (J.-I.P.); 2 Department of Genetics and Plant Breeding, Bangladesh Agricultural University, Mymensingh 2202, Bangladesh; 3 Center for Horticultural Seed Development of Golden Seed Project (GSP), Suncheon National University, 255 Jungang-ro, Suncheon, Jeonnam 57922, Korea; jihee0830@scnu.ac.kr; 4 University-Industry Cooperation Foundation, Suncheon National University, 255 Jungang-ro, Suncheon, Jeonnam 57922, Korea; htkim@sunchon.ac.kr

**Keywords:** interspecies hybridization, orange color, cabbage, Chinese cabbage

## Abstract

Vegetables in *Brassica* are some of the world’s most commonly cultivated plants and have a wide range of consumable plant organs. Improvement of this group of vegetables is limited at the species level due to limited genetic variability. Interspecies hybridization could be a powerful alternate tool for broadening the genetic variability of target traits. Embryo rescue technique is necessarily practiced in interspecies hybridization for protecting embryos from premature abortion. However, its success depends on the age of ovaries, shape of embryos, and the effect of female genotype. In this study, we carried out a wide range of interspecies crossing for introgressing target traits (orange/yellow color in cabbage and anthocyanin in Chinese cabbage) and optimizing the appropriate age of ovaries, the shape of embryo, and the suitable genotypes of such crosses. We observed that 15 DAP (days after pollination) was the best for embryo rescue in the diploid-diploid (*Brassica rapa* × *B. oleracea*) crosses, while 20 DAP was optimum for amphidiploid-diploid (*B. napus*/*B. juncea* × *B. rapa*) crosses. Cotyledonary shape of embryos and genotypes of amphidiploid species were the best for successful plant regeneration in interspecies crosses. We successfully selected plants with desired orange/yellow inner leaves for cabbage and higher anthocyanin in Chinese cabbage. The results of this study have the potential to be applied for the efficient production of interspecific hybrids and to develop *Brassica* vegetables with new traits, which could have potential for the enrichment of the human diet.

## 1. Introduction

*Brassica* is a genus, out of 51 of the tribe Brassiceae, belonging to the family Brassicaceae, containing 38 different species [1,2]. The Brassicaceae family includes a number of the world’s most commonly cultivated vegetables and oilseed species. *Brassica* vegetables comprise a large number of taxonomically closely related but morphologically diverse plants. These species have been cultivated over centuries and extensively crossed and hybridized to develop many cultivars worldwide from the tropics to the Arctic Circle [3].

The genome structures of *Brassica* were shaped by whole-genome triplication followed by extensive diploidization, which plays an important role in the speciation and morphotype diversification of *Brassica* plants [4]. The six species involved in “U-Triangle”, such as the diploid *B. rapa* (AA genome), *B. nigra* (BB), and *B. oleracea* (CC) as well as the allotetraploid species *B. juncea* (AABB), *B. napus* (AACC), and *B. carinata* (BBCC), are economically important and cultivated as vegetables, condiments, and sources of oilseed [2]. The amphidiploid species are the result of interspecies hybridization followed by natural chromosome doubling of the diploid species of relevant genome donor of “U-Triangle”. Improvements in *Brassica* are largely governed by the exploitation of the naturally occurring genetic variations in the species which are utilized in hybridization programs. Scientists are still trying to produce new morphotypes of *Brassica* or transfer traits of interest from one species to others following interspecific hybridization.

Interspecific hybridization is a powerful tool that plays an important role in transferring valuable traits between species of commercial interest [5,6,7]. Breeders have often attempted to hybridize close relatives of *Brassica* for introgressing novel traits in new and improved varieties. However, such interspecific hybridizations are often difficult as a result of pre- and post-fertilization barriers as well as the abortion of hybrid embryos [8,9]. With the advancement of biotechnological tools, especially in vitro culture and embryo rescue, it is now possible to achieve interspecific hybrids [10,11]. The rescuing of inherent weak, immature hybrids is also an important aspect of breeding programs in order to avoid genetic degeneration [12].

In *Brassicaceae*, due to the limited availability of variations within the species level, interspecific hybridizations are necessarily conducted for the modification of ecotypes with disease and insect tolerance and/or to alter nutritional quality [13]. In addition, embryo rescue followed by interspecific hybridization has been used for introgressing beneficial agronomic traits from wild species to cultivated crops [14,15,16]. Embryo rescue involves isolating and growing an immature or mature zygotic embryo under sterile conditions on an aseptic nutrient medium with the goal of obtaining a viable plant [17,18].

*B. napus* has been resynthesized successfully from the progenitor diploid species (*B. oleracea* and *B. rapa*) by interspecific hybridization and embryo rescue [19]. Interspecific hybrids were produced from the cross between *B. juncea* and *B. campestris*, and between *B. juncea* and *B. napus* employed embryo and ovary cultures [20,21,22]. The embryo rescue technique is a potential means in which to regenerate haploid plants in order to achieve a reduced breeding cycle [23]. In *Brassica*, the success of embryo rescue depends largely upon the maturation stage of embryos, composition of the medium, and, to some extent, on the genotype [24]. There are two basic growth stages in embryo development: heterotrophic and autotrophic [25]. In the heterotrophic stage, embryo development is controlled by the nutrient supply from the endosperm/cotyledon, while the later stage is not dependent on such nutrition supply [25]. In *Brassica*, it is recommended that embryo rescue techniques are applied as early as 10 to 30 days after pollination [18,26,27]. However, most of the results are reported on oilseed type *Brassica* plants, where *B. napus* or *B. campestris* were used as female parents and crossed with either wild or related taxa for their improvement. Limited reports are available on interspecific cross and embryo rescue in vegetable type *Brassica* plants for the purpose of their enrichment of secondary metabolites. 

Anthocyanin and β-carotenes are two secondary metabolites and water-soluble pigments that are widely distributed in plants, accumulating in the leaves, petals, sepals, and fruits to yield purple and/or red to yellow-orange coloration [28,29,30]. Eating foods enriched with secondary metabolites might reduce inflammation and protect against certain cancers, cardiovascular, neurodegenerative, and various age-related diseases [31,32,33,34]. Red cabbage has a good amount of anthocyanins, while anthocyanins are lacking in Chinese cabbage. In contrast, recent reports have suggested the presence of orange/yellow (probably β-carotene) coloration in the leaves of Chinese cabbage [35,36,37], but such coloration is lacking in cabbage.

Therefore, interspecies hybridization with the aim to introgress such anthocyanin content in Chinese cabbage and orange/yellow coloration in cabbage was attempted. The main objectives of our study were to evaluate the crossability among *B. rapa* (AA), *B. juncea* (BBCC), resynthesized *B. napus* (AACC), and *B. oleracea* (CC) genotypes for enrichment of β-carotene and anthocyanins. In addition, this study sough to identify the optimum developmental stage and age of embryos for rescue in interspecific crosses and calculate the efficacy of embryo rescue.

## 2. Materials and Methods

### 2.1. Plant Materials

A group of β-carotene-enriched Chinese cabbage (*B. rapa* subsp. *pekinensis*) cultivars, namely Orange queen (with *critso* mutant), β-Flash (with—*orange color* (*OR*) mutant), and BRP-K-42 (natural yellow inner leaves), were crossed with eight white cabbage (*B. oleracea* var. capitata) lines: ASC58, ASC61, ASC82, ASC47, ASC48, ASC117, J177, and J129 with the aim of transferring β-carotene (yellow/orange color) genes from Chinese cabbage to cabbage. For enriching anthocyanin in Chinese cabbage, the cultivars Anticancer, CR-langgawang, and CR-jungumi were crossed with anthocyanin-enriched amphiploid species *B. juncea* (cultivar Rogusa) and resynthesized *B. napus* line (Rs035; is the product of chromosome doubling followed by the interspecific cross between an inbred red colored Chinese cabbage line ‘Asia’ and a red cabbage cultivar ‘Rubea’). Backcross 1 (BC_1_) and backcross 2 (BC_2_) populations were developed by backcrossing with colchiploid F_1_ and cabbage lines for yellow/orange color transfer to cabbage, while Chinese cabbage lines were backcrossed for their anthocyanin enrichment.

### 2.2. Interspecific Hybridization Followed by Hand Emasculation

Interspecific hybridization was performed between Chinese cabbage and cabbage by using the Chinese cabbage cultivars as female parent, whereas Chinese cabbage cultivars (Anticancer, CR-langgawang, and CR-jungumi) were used as pollen parent in the crosses with amphiploid species (*B. juncea* and *B. napus*). Mature flower buds, which were ready to bloom within next 2–3 days, were selected for emasculation. Anthers of the selected flower buds were removed carefully with clean forceps by opening the buds while avoid the bursting of any anthers. Fresh pollen grains were collected from the recently opened flower of the target pollen parent and placed on the stigma of emasculated flower buds by dusting method. Two hundred buds were pollinated for each of the cross combinations. The pollinated buds were immediately covered with a crossing bag in order to avoid any unwanted pollination. Bags were removed carefully 5–7 days after pollination.

### 2.3. Embryo Rescue and Plant Regeneration

The siliquae were collected 10, 15, 20, 25, and 30 days after pollination. The siliquae were surface sterilized with 70% ethanol for 3 min, rinsed with sterile distilled water under aseptic condition, and then sterilized with 1% sodium hypochlorite (NaOCl; bioWORLD, Dublin, OH, USA) mixed with a drop of Tween-20 and left to sit for 10 min, and then finally rinsed three times with sterilized distill water. Well-developed ovules containing the embryos were excised and isolated from the siliquae, which was opened longitudinally with the help of a scalpel. The isolated ovules were cultured on petri-dishes containing Murashige and Skoog (MS) medium [38] with 30 g/L sucrose, 9 g/L agar, with the pH adjusted to 5.8. The cultured embryos were maintained under fluorescent light at 13 h/11 h (light/dark) with 23 ± 2 °C. Differently shaped embryos were generated from ovule after 8–10 days of culture. Embryos were germinated and produced shoot within 15–20 days of inoculation. True to type plants with sufficient root systems were achieved from the sub-cultured shoots on MS basal salts within 35–40 days of inoculation.

### 2.4. Chromosome Doubling, Selection of F_1_ and Amphidiploids Plants

Well-rooted plants were taken away from MS medium and washed to remove the medium attached to roots; thereafter, plants roots were submerged in 0.1% colchicine (Merck, Darmstadt, Germany) and kept for 4 h in the fluorescent light with 1000 lux. Plant roots were washed three times with fresh running tap water in order to remove colchicine from the roots; finally, plants were planted in multi-hole trays containing coco-peat soil, hardened by maintaining high humidity for one week, and then transferred to a greenhouse. Successfully crossed hybrid plants were identified by PCR amplification of A-, B-, and C-genome-specific bands from the DNA of plant leaves by using the Conserved Ortholog Set (COS) marker COS1078 [39].

DNA was isolated from 1 g of the leaves of the parental lines and the interspecies hybrids plants following the protocol described by Ishizawa et al. [40] with slight modifications. PCR was carried out for detecting the amplification of COS and *OR* mutant using 20 µL of PCR mix, which contained 1 μL DNA, 1 μL of both the forward and reverse primer (10 pmol), 8 μL Prime TaqPremix (2×) (GENETBIO Inc., Daejong, Korea), and 9 μL ultra pure H_2_O. The PCR protocol was fixed as follows: initial denaturation at 72 °C for 3 min, then 35 cycles of denaturation at 72 °C for 30 s, annealing at 56 °C for 30 s, elongation at 72 °C for 3 min, and for the last step, the final elongation at 72 °C for 5 min. Finally, the PCR was cooled down and kept at 4 °C. The *OR* mutant, responsible for orange color, was confirmed by using the *OR* mutant-specific primers [41].

The genotypes of the allopolyploid plants with diploid chromosomes were confirmed using CyFlow Ploidy Analyzer and 4′,6-diamidino-2-phenylindole (DAPI) solution (Sysmex, Norderstedt, Germany). Next, 1 cm^2^ of young leaf was taken from 1-month-old plants, finely chopped by a single edge blade (Dorco, Seoul, Korea), and placed in 500 µL DAPI solution. The DAPI solution containing the leaf cells was filtered using 30 µm CellTrics disposable filters (Sysmex, Goerlitz, Germany). The filtrate was mixed with 2.0 mL Cystain@ UV precise P staining buffer, placed into a 3.5-mL tubes, and loaded in the CyFlow Ploidy Analyzer (Cysmex Partec, Leipzig, Germany) panel. The filtrate injection rate was fixed 0.3 µL/s and the sharp pick of the cell line spectra was considered for detecting the ploidy level. 

### 2.5. Cytological Observation

Anthers from the flower buds of embryo-rescued plants were used for cytological study. Inflorescence with immature flower buds was fixed in Carnoy’s solution (60% ethanol, 30% chloroform, 10% glacial acetic acid, and 1 g of ferric chloride) and maintained at 15 °C for 3 h. After fixation, one flower bud that was 3–4 mm in size was opened by forceps and its anthers were collected and placed on a clean microscopic slide. Thereafter, one anther was placed into a drop of 0.2% acetocarmine solution on a clean slide and squashed gently by the back of the needle. Visible anther wall was removed by needle and a coverslip (1.5 × 1.5 cm) was placed on the squashed anther; thereafter, chromosomes were stained and spread by tapping and warming the slide on a flame (sprit lamp). After staining, the chromosome spreads were observed under a light microscope (Leica DM750; Wetzlar, Germany) for chromosome counting [42].

For the pollen sterility test, anthers were dissected from the flower buds and stained with 0.2% acetocarmine, then pollen sterility was recorded as the ratio of fertile (dark stained) and sterile (pale yellow stained) pollen grains observed under the light microscope (Leica). 

### 2.6. Estimation of Total Anthocyanin and Statistical Analysis

The total anthocyanin content was determined following a previously described protocol [43] with some modifications. Three leaves of each genotype were frozen in liquid nitrogen and ground into powder, then 100 mg of each sample was transferred into an Eppendorf tube containing 1 mL acidic methanol (1% HCl, *w*/*v*). Samples were mixed overnight at room temperature by shaking at 50 rpm in the dark. After that, the mixtures were centrifuged at 12,000× *g* for 10 min. The supernatants were collected and the absorbance of each sample was determined at 530 and 657 nm wavelengths. The total anthocyanin content was computed using the following equation: Q_Anthocyanins_ = (A_530_ − 0.25 × A_657_) × FW^−1^, where Q_Anthocyanins_ refers to the total anthocyanin content, A_530_ refers to the absorption at 530 nm, A_657_ refers to the absorption at 657 nm, and FW refers to the fresh weight of the leaf samples (g). The total anthocyanin content was quantified from three replicates of each biological sample. The anthocyanin content was analyzed using one-way ANOVA and Tukey’s pair-wise comparisons in Minitab v.17 (Minitab Inc., State College, PA, USA).

## 3. Results

### 3.1. Days after Pollination (DAP) Contributes to the Success of Embryo Rescue

**Figure 1:**
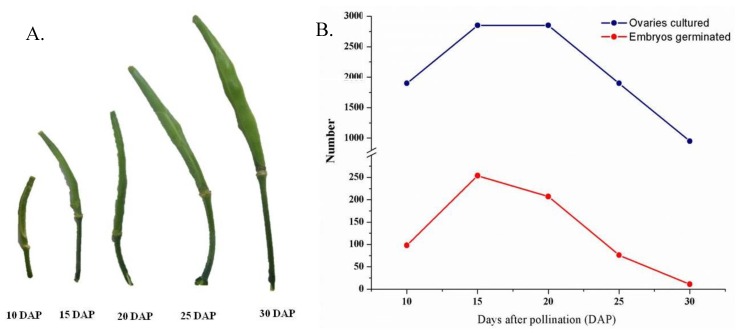
(**A**) Developmental stages and size of the siliquae on different days after pollination (DAP) used for isolating ovaries; (**B**) Number of successfully isolated ovaries and total number of embryos germinated on that particular DAP for all the interspecies crosses.

**Figure 2:**
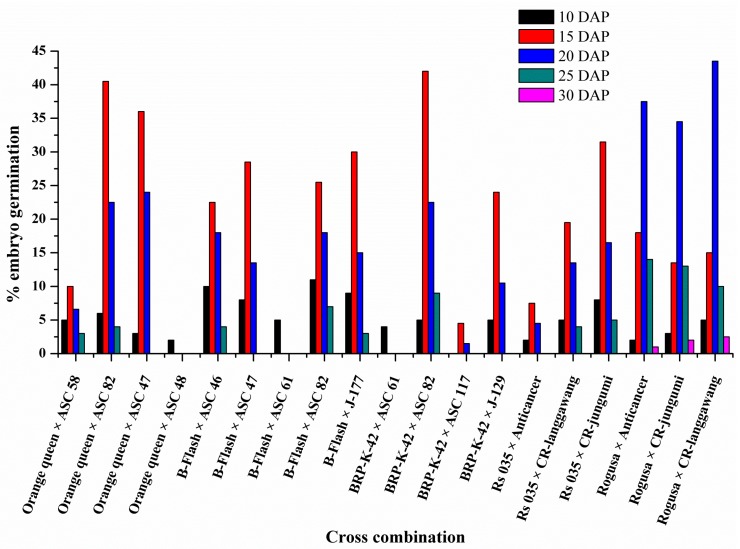
Efficacy of the embryo germination from the isolated ovaries at different days after crossing in different cross combinations.

The ovaries of the interspecies crosses isolated and cultured at 10, 15, 20, 25, and 30 DAP on the regeneration medium showed various degrees of embryo germination capability. However, isolation of ovaries was tedious at 10 DAP due to their small size (Figure 1A). Therefore, only a small number of ovaries could be isolated at 10 DAP. In contrast, most of the ovaries dried out at 25 DAP and onwards, which also led to a smaller number of such ovaries being successfully isolated (Figure 1B). Ovaries isolated at 15 DAP had a higher tendency of embryo germination for most of the crosses (Figure 1B). In interspecies crosses between *B. juncea* (Rogusa) and *B. rapa* parents (Anticancer, CR-langgawang, and CR-jungumi), the highest number of germinated embryos were obtained at 20 DAP (Figure 2 and Table S1). The ovaries from the cross Rogusa × CR-langgawang were more effective in terms of embryo germination when isolated at 20 DAP compared to other time points, while isolated ovaries of the crosses BRP-K-42 × ASC 82 and Orange queen × ASC 82 showed better embryo germination performance at 15 DAP compared to other time points. No embryo germination was achieved for the ovaries isolated at 30 DAP in most of the crosses, with the exception of the crosses made with Rogusa. Some embryo germination occurred from the ovaries isolated at 25 DAP in the crosses of resynthesized *B. napus* (Figure 2 and Table S1), indicating that 15 DAP was the most suitable stage of embryo development for rescuing in diploid-diploid crosses. However, embryos could be successfully rescued up to 20–25 DAP in the crosses made with the genotypes of amphidiploid species. In terms of cross combination, the highest rate of embryo germination reached was 43.5% in the cross Rogusa × CR-langgawang at 20 DAP followed by 42% and 40.5% in the crosses BRP-K-42 × ASC 82 and Orange queen × ASC 82, respectively, at 15 DAP (Figure 2 and Table S1).

### 3.2. Genotypes Contribute to the Success of Embryo Rescue of Interspecies Crosses

**Figure 3:**
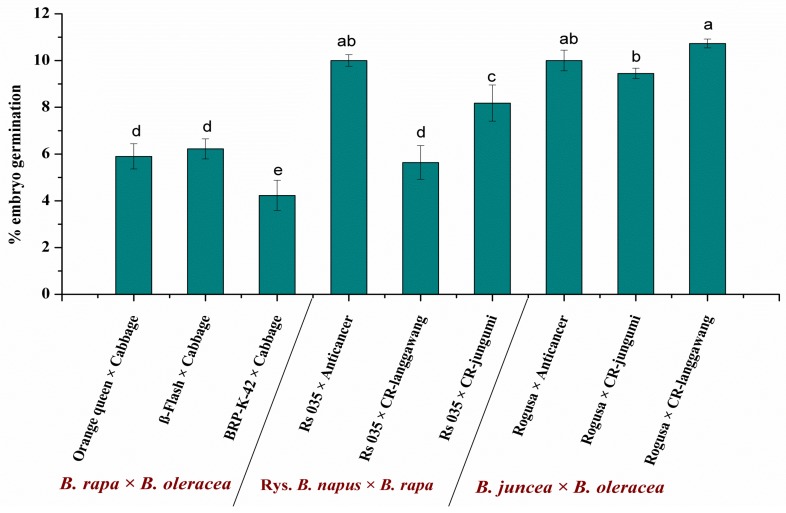
The rate of embryo germination (%) obtained by different cross combinations of ovary culture of interspecies crosses between diploid species and amphidiploid and diploid species in *Brassica*. The lettering indicates the significance of mean comparison according to Tukey’s method at *p* < 0.05.

Significant variation was observed among the crosses in response to embryo germination from isolated and cultured ovaries. The best response was found in the cross Rogusa × CR-langgawang, with embryo germination reaching 10.73%, followed by the crosses Rogusa × Anticancer and Rs 035 × Anticancer, each of which showed 10% embryo germination (Figure 3 and Table S2), while the cross BRP-K-42 × Cabbage resulted in 4.23% of embryo germination. Crosses made with amphiploid parents showed higher rates of embryo germination than the crosses between diploid species (Figure 3 and Table S2) This might be due to the presence of the counterpart of the A-genome donor parent (*B. rapa*; AA) in the amphiploid parents (*B. juncea*; AABB and Rys. *B. napus*; AACC). The presence of the counterpart of the donor parent assured high chromosome pairing among the homologs of the A-genome and also assured the production of a high number of embryos.

### 3.3. Effect of Embryo Shape on Plant Regeneration from Rescued Embryo

**Figure 4:**
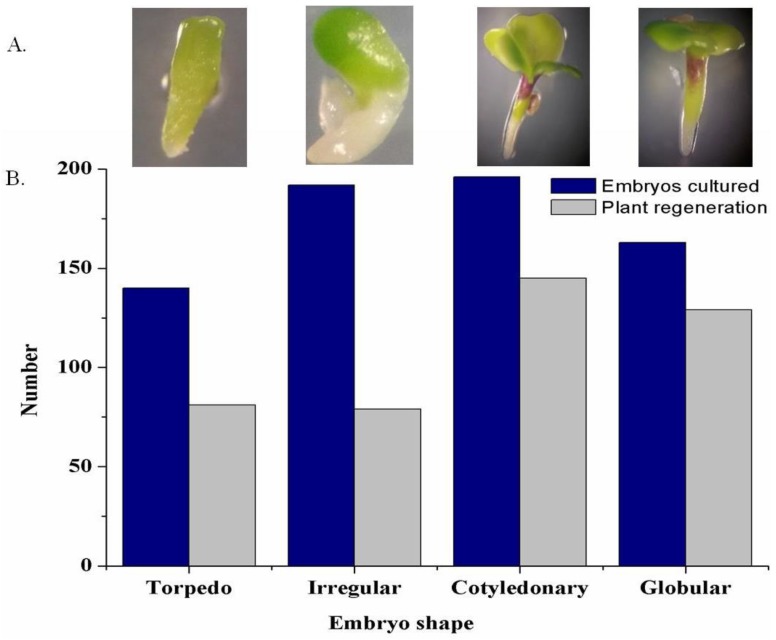
Types of embryo formation and regeneration ability. (**A**) Different shapes of the embryos developed from the rescued ovaries of the interspecies crosses; (**B**) The plant regeneration ability of the cultured embryos isolated from the interspecies crosses.

We investigated how the plant regeneration rate corresponded to the shape of the embryos, which were developed from in vitro ovary cultured on MS media. This was done in order to know whether embryo shape influenced the rate of plant regeneration. Four distinct types of embryos, namely torpedo, irregular, cotyledonary, and globular, were developed from the isolated and cultured ovaries of interspecies crosses at different days (Figure 4A). The embryos were most commonly cotyledonary in shape followed by irregular, globular, and torpedo in shape. By contrast, the highest plant regeneration rates were obtained from the cotyledonary-shaped embryos followed by globular-, torpedo-, and globular-shaped embryos (Figure 4B). The cotyledonary-shaped embryos showed significantly higher rates of plant regeneration in the interspecies crosses between two parents of diploid species. Moreover, plant regeneration was significantly higher for globular-shaped embryos in the crosses between parents of amphidiploid and diploid species (Table 1).

### 3.4. Confirmation of Embryo-Rescued Hybrid Plants

**Figure 5:**
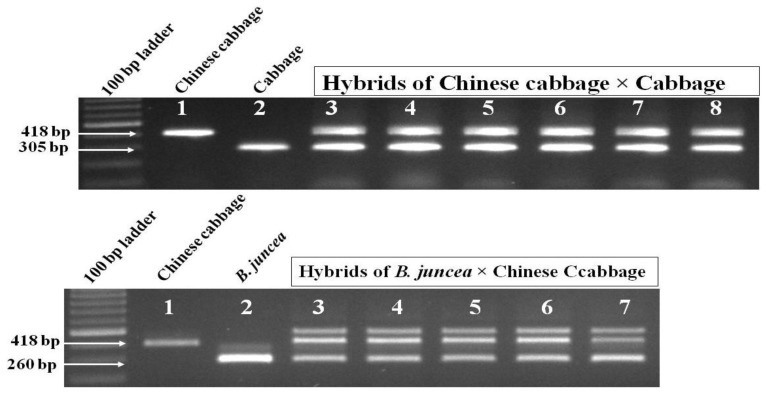
Gel picture of genome-specific PCR amplicons for A-, B-, and C-genome in the hybrid plants of the crosses between Chinese cabbage and cabbage (upper panel) as well as between *B. juncea* and Chinese cabbage (lower panel). In the case of hybrids of the *B. juncea* × Chinese cabbage, the upper two bands could be due to the hybridity bands of the hybrid *B. juncea* parent.

**Figure 6:**
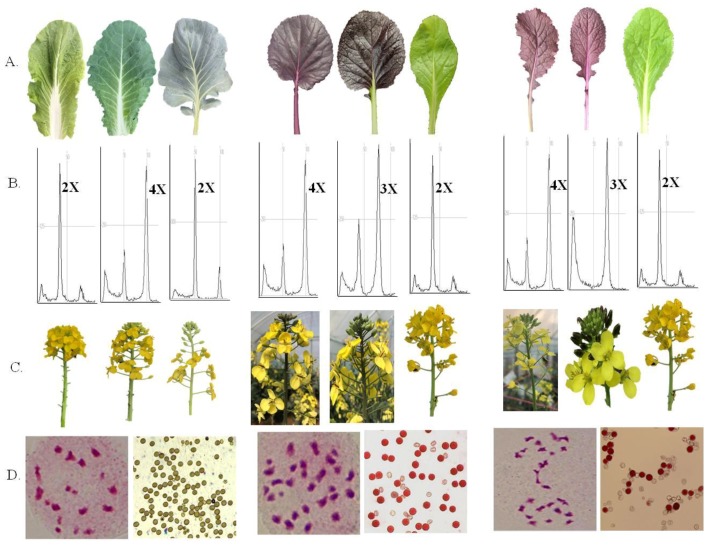
Confirmation of hybrid plants of the interspecies crosses involving diploid species to diploid species (*B. rapa* × *B. oleracea*) and amphidiploid species to diploid species (*B. napus* × *B. rapa*) and (*B. juncea* × *B. rapa*); (**A**) Intermediate leaf morphology in hybrids between their parents; (**B**) Flowcytometry peaks of the hybrids and their parents; (**C**) Intermediate inflorescence type in hybrids of their parents; and (**D**) Meiotic chromosome number and behavior in metaphase 1 (left) along with the pollen sterility status (right) of the hybrid plants.

A wide range of interspecies crosses among genotypes of different *Brassica* species (detailed in materials and methods) was done. On the 10th, 15th, 20th, 25th, and 30th day after pollination, the young siliquae were harvested, their surfaces were sterilized, and then their ovules were dissected out and grown in MS medium. Seedlings were developed within 20–30 days after dissection from siliquae. PCR amplification of COS1078 marker using the DNA of hybrid plants confirmed the hybridity of *B. rapa* × *B. oleracea* and *B. juncea* × *B. rapa* (Figure 5). In addition to this, the intermediate type leaf morphology of the parents also confirmed the formation of hybrid plants, such as *B. rapa* × *B. oleracea*, *B. napus* × *B. rapa*, and *B. juncea* × *B. rapa* (Figure 6A). The hybrid plants of the cross between lines/cultivars of diploid species (*B. rapa* × *B. oleracea*) were treated with colchicines for chromosome doubling. The flowcytometry analysis showed peaks at tetraploid (4×) position for colchiploids of the crosses of *B. rapa* × *B. oleracea*. The peaks were in triploid (3×) position for the crosses of *B. napus* × *B. rapa* and *B. juncea* × *B. rapa* (Figure 6B). Morphologically, the inflorescences of the hybrid plants of the crosses were intermediate between the parents (Figure 6C). The hybrid plants were male sterile and produced no pollen of the crosses of diploid species (*B. rapa* × *B. oleracea*) before colchicine treatment. They produced functional pollen in the colchiploid after colchicine treatment. In contrast, the crosses between amphidiploid and diploid species showed partial pollen sterility (Figure 6D). In the case of the crosses of *B. juncea* × *B. rapa*, pollen sterility was higher than the crosses of *B. napus* × *B. rapa*. Chromosome analysis from the flower bud cells revealed that the hybrid cells contained 19, 29, and 28 chromosomes for *B. rapa* × *B. oleracea* (colchiploid), *B. napus* × *B. rapa*, and *B. juncea* × *B. rapa*, respectively (Figure 6D). In addition, one chromosome bridge with four chromosomes was observed in the case of *B. juncea* × *B. rapa*.

### 3.5. Determination of Orange Color and Anthocyanin in BC1 and BC2 Progeny

**Figure 7:**
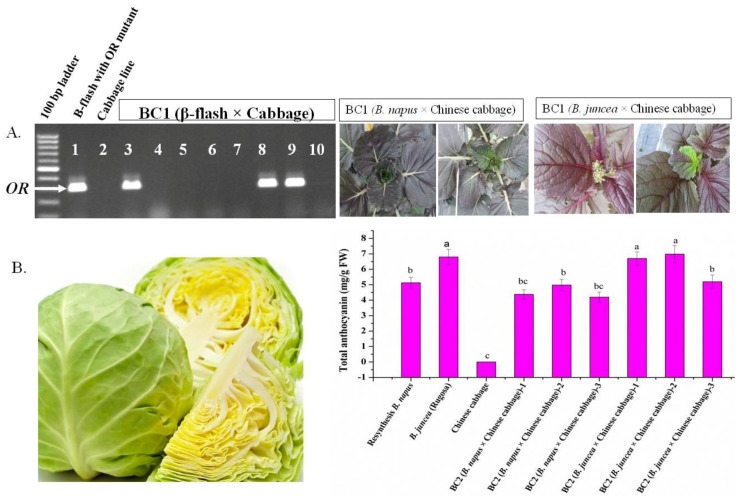
Variation in target gene segregation and morphology of the target traits in the backcross 1 (BC1) and backcross 2 (BC2) generation of the interspecies crosses. (**A**) Segregation of the target *OR* mutant in BC1 progeny (left panel) and morphological variation in anthocyanin content; (**B**) Presence of yellow/orange color in inner cabbage leaves (left panel) and the variation of total anthocyanin content (right panel) in the selected plants of the BC2 of the interspecies crosses. The lettering on bar graph indicates the significance of mean comparison according to Tukey’s method at *p* < 0.05.

BC1 and BC2 progenies were developed to transfer the recipient parent’s genome to the hybrid background; for example, cabbage parents were crossed back with the hybrids of *B. rapa* × *B. oleracea* and Chinese cabbage parents were crossed back with the hybrids of amphidiploid × *B. rapa*. All of the BC1 plants were fertile, except the cross between β-flash (CMS) and cabbage. The BC1 plants segregated for *OR* mutant (please see the left panel of Figure 7A) and showed variation in anthocyanin content (please the right panel of Figure 7A) for the crosses of β-flash × cabbage and amphidiploid × *B. rapa*, respectively. Morphologically, the BC2 plants bore a stronger resemblance to the recipient parents (for example, more cabbage-like for cabbage recipient parents or more Chinese cabbage-like for Chinese cabbage recipient parents). Some of the plants of the BC2 progenies showed morphological features that indicated the successful introgression of target traits, such as yellow/orange inner leaves in cabbage and the total anthocyanin content in Chinese cabbage, but when compared to their parents the differences were non-significant (Figure 7B). The phenotypes of these hybrids presented evidence of the successful introgression of the secondary metabolites (orange/yellow color and anthocyanin) through interspecies hybridization. These interspecific hybridizations showed remarkable potential for improvement of cabbage and Chinese cabbage, which are both highly valued vegetables.

## 4. Discussion

*Brassica* species and allies contain useful genes that could be incorporated into breeding programs through interspecies hybridization. Interspecies hybridization is an important tool for the introgression of target traits into commercial cultivars [14,44]. It is often limited by pre- and post-fertilization barriers as well as the abortion of hybrid embryos [8,9]. To overcome this limitation, embryo rescue techniques are widely used in breeding programs for rescuing inherent weak, immature hybrids in order to avoid degeneration [12]. The success of embryo rescue depends on several factors, such as the age of the embryo, shape of embryo, genotypes, and media composition [23]. The experiment reported here showed that through ovary culture, embryos were produced and plantlets were regenerated from different types of interspecies crosses. It was observed that the embryo isolation time (days after pollination; DAP), cross combination, embryo shape, and genotypes were crucial factors influencing the rate of plant regeneration. The ovaries cultured on the 10th, 15th, 20th, 25th, and 30th day after pollination showed wide variations in success. The results indicated that the ovaries isolated at 15 DAP from the cross between parents of diploid species (*B. rapa × B. oleracea*) had a higher tendency of embryo formation (Figure 2 and Table S1). In addition, the ovaries isolated at 20 DAP from the cross between parents of amphidiploid and diploid species (*B. napus*/*B. juncea × B. rapa*) formed higher numbers of embryos. However, embryos could not be formed using the ovaries from 30 DAP. Similar results were found in the interspecific hybridization of *B. napus* × *B. oleracea* [45] and *B. napus* × *B. juncea* [46].

It was observed that there was significant variation among the response of the genotypes to embryo formation after interspecific hybridization and embryo rescue (Figure 3 and Table S2). In order to introgress the orange/yellow color trait, the isogenic lines of Chinese cabbage were used, whose adaptation to our growing condition was not so good, while locally adapted resynthesis *B. napus* and *B. juncea* lines/cultivars were used as a source of anthocyanin. Apart from this poor local adaptation, the cytological interactions might affect the growth, vigor, and compatibility of the hybrid ovaries of the crosses of *B. rapa* × *B. oleracea*. As a result, the efficiency of the crosses in terms of embryo formation varied within the cross combinations. However, once the embryos were germinated, their rate of plant regeneration was similar to the cross combinations of *B. napus* × *B. rapa* and *B. juncea* × *B. rapa* (Table 1), indicating the potential of using diverse germplasms in interspecific hybridization for trait introgression. Our results are in agreement with the observations associated with the resynthesizing of *B. napus* from interspecific hybridization between *B. rapa* and *B. oleracea* [23].

The shape of the embryos from cultured ovaries is crucial for plant regeneration in interspecific hybridization. Four types of embryo shape (globular, torpedo, cotyledonary, and irregular) were recorded. The present experiment showed that the types of embryo had influence on plant regeneration. Considering all types of interspecies crosses, the cotyledonary-shaped embryos presented a higher rate of plant regeneration compared to the other embryo types (Figure 4 and Table 1). The results were consistent with other reports of embryo rescue in different cross combinations of Chickpea and its wild relatives [47] and in banana [48].

The PCR amplicon of COS1078 marker, leaf morphology, flowcytometry peaks, inflorescence shape, and cytological analysis demonstrated that hybridization was successful (Figure 6A–D). The results showed that the chromosome number of all plants tested was 19, 29, and 28 for the crosses of *B. rapa* × *B. oleracea*, *B. napus* × *B. rapa*, and *B. juncea* × *B. rapa*, respectively. Cytological identification and the flow-cytometer analysis were able to directly determine the chromosome number (ploidy levels) of regenerated plants from the fresh leaves [49,50].

Chromosome doubling was attempted in the regenerated plants of the crosses of *B. rapa* × *B. oleracea* but did not do for the plants of the crosses of *B. napus*/*B. juncea* × *B. rapa*. Thereafter, plants were grown in the field for use as female parents in a backcrossing programme. The colchiploid plants of the cross *B. rapa* × *B. oleracea* did not show pollen sterility because of regular meiosis, while the interspecies hybrids of amphidiploids × diploid species showed different degrees of pollen sterility (Figure 6D), which might be the result of the uneven recombination of non-homologous chromosome segments. Among the hybrids of amphidiploid × diploid species, the hybrid plants of *B. juncea* × *B. rapa* showed higher pollen sterility due to presence of a chromosomal bridge in meiosis-1, which lowered the chances of separation of the homologous chromosomal segment. Sequencing results showed that the A-, B-, and C-genomes have many common homologous segments, therefore offering opportunities of recombination among them [51,52]. The BC1 progenies of the interspecies hybrids showed a wide range of variation at the molecular and morphological level. These variations are expected because *Brassica* genomes passed through a triplication event during their evolution in order to arrive at their final shape and subgenomes were produced in the process [53], which show plasticity for non-homologous recombination among them. BC2 progenies had less variation compared to BC1 because of enriching recipient genome.

Commercially important agronomy traits, like male sterility and biotic and abiotic stress tolerance have been introgressed to cultivars from relative taxa or wild species by interspecies hybridization [54,55]. However, variation within the species has sharply declined due to high selection pressure to produce homogenous commercial cultivars. Given that high valued crop innovation is prioritized, there is a great incentive to do interspecies hybridization and subsequent backcrossing for introgressing new traits.

## 5. Conclusions

This study shows that success in interspecific hybridization regarding plant regeneration is dependent on age of ovaries, shape of embryos, and the genotypes involved in such crosses. We found 15 DAP and 20 DAP are the optimum ages of ovaries for diploid-diploid and amphidiploid-diploid crosses, respectively. In addition, we found that the cotyledonary-shaped embryos and amphidiploid female parents are important factors for obtaining high frequency regeneration. The results of this study have the potential to be applied for the efficient production of interspecific hybrids in *Brassica* vegetables. In addition, the present study has generated new traits in *Brassica* vegetables that could have the potential to help enrich the human diet.

## Figures and Tables

**Table 1:** The rate of plant regeneration (%) obtained by different shapes of embryos developed from different cross combinations of interspecies crosses between diploid species and amphidiploid and diploid species in *Brassica*. The lettering indicates the significance of mean comparison according to Tukey’s method at *p* < 0.05.

Cross Type	Cross Combination	Embryo Shape	No. of Embryos Cultured	No. of Plants Regenerated	Rate of Plant Regeneration (%)
*B. rapa* × *B. oleracea*	Orange queen × Cabbage	Torpedo	30	18	60.00 b
		Irregular	25	10	40.00 c
		Cotyledonary	35	29	82.80 a
		Globular	40	33	82.50 a
	β-Flash × Cabbage	Torpedo	40	27	67.50 b
		Irregular	55	22	40.00 c
		Cotyledonary	48	36	75.00 b
		Globular	28	20	71.50 a
	BRP-K-42 × Cabbage	Torpedo	20	11	55.00 b
		Irregular	25	12	48.00 c
		Cotyledonary	28	20	71.43 a
		Globular	20	15	75.00 a
*Rys. B. napus* × *B. rapa*	Rs 035 × Anticancer	Torpedo	10	7	70.00 b
		Irregular	15	9	60.00 c
		Cotyledonary	12	9	75.00 b
		Globular	18	15	83.33 a
	Rs 035 × CR-langgawang	Torpedo	8	3	37.50 c
		Irregular	7	3	42.86 c
		Cotyledonary	10	7	70.00 b
		Globular	6	5	83.33 a
	Rs 035 × CR-jungumi	Torpedo	7	4	57.14 c
		Irregular	10	3	30.00 d
		Cotyledonary	16	12	75.00 b
		Globular	12	10	83.33 a
*B. juncea* × *B. rapa*	Rogusa × Anticancer	Torpedo	10	4	40.00 b
		Irregular	20	8	40.00 b
		Cotyledonary	15	12	80.00 a
		Globular	10	8	80.00 a
	Rogusa × CR-jungumi	Torpedo	11	5	45.45 b
		Irregular	15	6	40.00 b
		Cotyledonary	12	10	83.33 a
		Globular	14	11	78.57 a
	Rogusa × CR-langgawang	Torpedo	4	1	25.00 b
		Irregular	20	6	30.00 b
		Cotyledonary	20	16	80.00 a
		Globular	15	12	80.00 a

## Supplementary Materials

The following are available online at http://www.mdpi.com/2223-7747/7/4/99/s1, Table S1: Number of ovaries isolated from different interspecies crosses in *Brassica* species with their success in embryo germination from the isolated ovaries, Table S2: The rate of embryo germination (%) obtained by different cross combinations of ovary culture of interspecies crosses between diploid species and amphidiploids and diploid species in *Brassica*, lettering indicates the significance of mean comparison in Tukey’s method at *p* < 0.05.
