# Supplementary material for: Developmental Stage and Shape of Embryo Determine the Efficacy of Embryo Rescue in Introgressing Orange/Yellow Color and Anthocyanin Genes of Brassica Species
Source: Plants (Basel). 2018 Nov 13;7(4):99. doi: 10.3390/plants7040099 (PMC6313868; doi:10.3390/plants7040099)
Supplement: Supplementary file 1 [file plants-07-00099-s001.zip › Supplementary Tables.doc]

**Supplementary Table S1.** Number of ovaries isolated from different interspecies crosses in *Brassica* species with their success in embryo germination from the isolated ovaries.

| Cross direction | Cross combination | DAP | No. of ovaries cultured | No. of germinated embryos | Rate of embryo germination (%) |
| --- | --- | --- | --- | --- | --- |
| *B. rapa* × *B. oleracea* | Orange queen × ASC 58 | 10 | 100 | 5 | 5 |
|  |  | 15 | 150 | 15 | 10 |
|  |  | 20 | 150 | 10 | 6.6 |
|  |  | 25 | 100 | 3 | 3 |
|  |  | 30 | 50 | 0 | 0 |
|  | Orange queen × ASC 82 | 10 | 100 | 6 | 6 |
|  |  | 15 | 150 | 27 | 40.5 |
|  |  | 20 | 150 | 15 | 22.5 |
|  |  | 25 | 100 | 4 | 4 |
|  |  | 30 | 50 | 0 | 0 |
|  | Orange queen × ASC 47 | 10 | 100 | 3 | 3 |
|  |  | 15 | 150 | 24 | 36 |
|  |  | 20 | 150 | 16 | 24 |
|  |  | 25 | 100 | 0 | 0 |
|  |  | 30 | 50 | 0 | 0 |
|  | Orange queen × ASC 48 | 10 | 100 | 2 | 2 |
|  |  | 15 | 150 | 0 | 0 |
|  |  | 20 | 150 | 0 | 0 |
|  |  | 25 | 100 | 0 | 0 |
|  |  | 30 | 50 | 0 | 0 |
|  | β-Flash × ASC 46 | 10 | 100 | 10 | 10 |
|  |  | 15 | 150 | 15 | 22.5 |
|  |  | 20 | 150 | 12 | 18 |
|  |  | 25 | 100 | 4 | 4 |
|  |  | 30 | 50 | 0 | 0 |
|  | β-Flash × ASC 47 | 10 | 100 | 8 | 8 |
|  |  | 15 | 150 | 19 | 28.5 |
|  |  | 20 | 150 | 9 | 13.5 |
|  |  | 25 | 100 | 0 | 0 |
|  |  | 30 | 50 | 0 | 0 |
|  | β-Flash × ASC 61 | 10 | 100 | 5 | 5 |
|  |  | 15 | 150 | 0 | 0 |
|  |  | 20 | 150 | 0 | 0 |
|  |  | 25 | 100 | 0 | 0 |
|  |  | 30 | 50 | 0 | 0 |
|  | β-Flash × ASC 82 | 10 | 100 | 11 | 11 |
|  |  | 15 | 150 | 17 | 25.5 |
|  |  | 20 | 150 | 12 | 18 |
|  |  | 25 | 100 | 7 | 7 |
|  |  | 30 | 50 | 0 | 0 |
|  | β-Flash × J-177 | 10 | 100 | 9 | 9 |
|  |  | 15 | 150 | 20 | 30 |
|  |  | 20 | 150 | 10 | 15 |
|  |  | 25 | 100 | 3 | 3 |
|  |  | 30 | 50 | 0 | 0 |
|  | BRP-K-42 × ASC 61 | 10 | 100 | 4 | 4 |
|  |  | 15 | 150 | 0 | 0 |
|  |  | 20 | 150 | 0 | 0 |
|  |  | 25 | 100 | 0 | 0 |
|  |  | 30 | 50 | 0 | 0 |
|  | BRP-K-42 × ASC 82 | 10 | 100 | 5 | 5 |
|  |  | 15 | 150 | 28 | 42 |
|  |  | 20 | 150 | 15 | 22.5 |
|  |  | 25 | 100 | 9 | 9 |
|  |  | 30 | 50 | 0 | 0 |
|  | BRP-K-42 × ASC 117 | 10 | 100 | 0 | 0 |
|  |  | 15 | 150 | 3 | 4.5 |
|  |  | 20 | 150 | 1 | 1.5 |
|  |  | 25 | 100 | 0 | 0 |
|  |  | 30 | 50 | 0 | 0 |
|  | BRP-K-42 × J-129 | 10 | 100 | 5 | 5 |
|  |  | 15 | 150 | 16 | 24 |
|  |  | 20 | 150 | 7 | 10.5 |
|  |  | 25 | 100 | 0 | 0 |
|  |  | 30 | 50 | 0 | 0 |
| Rys*. B. napus* × *B. rapa* | Rs 035 × Anticancer | 10 | 100 | 2 | 2 |
|  |  | 15 | 150 | 5 | 7.5 |
|  |  | 20 | 150 | 3 | 4.5 |
|  |  | 25 | 100 | 0 | 0 |
|  |  | 30 | 50 | 0 | 0 |
|  | Rs 035 × CR-langgawang | 10 | 100 | 5 | 5 |
|  |  | 15 | 150 | 13 | 19.5 |
|  |  | 20 | 150 | 9 | 13.5 |
|  |  | 25 | 100 | 4 | 4 |
|  |  | 30 | 50 | 0 | 0 |
|  | Rs 035 × CR-jungumi | 10 | 100 | 8 | 8 |
|  |  | 15 | 150 | 21 | 31.5 |
|  |  | 20 | 150 | 11 | 16.5 |
|  |  | 25 | 100 | 5 | 5 |
|  |  | 30 | 50 | 0 | 0 |
| *B. juncea* × *B. rapa* | Rogusa × Anticancer | 10 | 100 | 2 | 2 |
|  |  | 15 | 150 | 12 | 18 |
|  |  | 20 | 150 | 25 | 37.5 |
|  |  | 25 | 100 | 14 | 14 |
|  |  | 30 | 50 | 2 | 1 |
|  | Rogusa × CR-jungumi | 10 | 100 | 3 | 3 |
|  |  | 15 | 150 | 9 | 13.5 |
|  |  | 20 | 150 | 23 | 34.5 |
|  |  | 25 | 100 | 13 | 13 |
|  |  | 30 | 50 | 4 | 2 |
|  | Rogusa × CR-langgawang | 10 | 100 | 5 | 5 |
|  |  | 15 | 150 | 10 | 15 |
|  |  | 20 | 150 | 29 | 43.5 |
|  |  | 25 | 100 | 10 | 10 |
|  |  | 30 | 50 | 5 | 2.5 |

**Supplementary Table S2.** The rate of embryo germination (%) obtained by different cross combinations of ovary culture of interspecies crosses between diploid species and amphidiploids and diploid species in *Brassica*, lettering indicates the significance of mean comparison in Tukey’s method at *p < 0.05*.

| Cross type | Cross combination | No. of ovaries cultured | No. of embryo germinated | Rate of embryo germination (%) |
| --- | --- | --- | --- | --- |
| *B. rapa* × *B. oleracea* | Orange queen × Cabbage | 2200 | 130 | 5.9 d |
|  | β-Flash × Cabbage | 2750 | 171 | 6.22 d |
|  | BRP-K-42 × Cabbage | 2200 | 93 | 4.23 e |
| Rys. *B. napus* × *B. rapa* | Rs 035 × Anticancer | 550 | 55 | 10 ab |
|  | Rs 035 × CR-langgawang | 550 | 31 | 5.64 d |
|  | Rs 035 × CR-jungumi | 550 | 45 | 8.18 c |
| *B. juncea* × *B. rapa* | Rogusa × Anticancer | 550 | 55 | 10 ab |
|  | Rogusa × CR-jungumi | 550 | 52 | 9.45 b |
|  | Rogusa × CR-langgawang | 550 | 59 | 10.73 a |
